# Diagnostic accuracy of circular RNA for diabetes Mellitus: a systematic review and diagnostic Meta-analysis

**DOI:** 10.1186/s12920-023-01476-0

**Published:** 2023-03-08

**Authors:** Hojat Dehghanbanadaki, Pooria Asili, Abdolkarim Haji Ghadery, Maryam Mirahmad, Ali Zare Dehnavi, Amirhossein Parsaei, Hamid Reza Baradaran, Mobin Azami, Gustavo Jose Justo da Silva, Reza Parvan, Yousef Moradi

**Affiliations:** 1 Endocrinology and Metabolism Research Center, Endocrinology and Metabolism Clinical Sciences Institute, Tehran University of Medical Sciences, Tehran, Iran; 2 Department of Radiology, Advanced Diagnostic and Interventional Radiology Research Center (ADIR), Tehran University of Medical Sciences, Tehran, Iran; 3 Ageing Clinical & Experimental Research Team, Institute of Applied Health Sciences, University of Aberdeen, Aberdeen, UK; 4 Student Research Committee, Kurdistan University of Medical Sciences, Sanandaj, Iran; 5 Department of Molecular Medicine, Institute of Basic Medical Sciences, University of Oslo, Oslo, Norway; 6 Institute for Experimental Medical Research, Oslo University Hospital and University of Oslo, University of Oslo, Oslo, Norway; 7 Social Determinant of the Health Research Center, Research Institute for Health Development, Kurdistan University of Medical Sciences, Sanandaj, Iran

**Keywords:** circRNAs, Diabetes, Diagnosis, Biomarker, Meta-analysis

## Abstract

**Background:**

This study aimed to investigate the pooled diagnostic ability of circular RNA (circRNA) molecules for diabetes mellitus.

**Methods:**

We searched PubMed, Scopus, and Web of Science for relevant studies. A total of 2070 participants, including 775 diabetic patients and 1295 healthy individuals, from five studies were included in this meta-analysis. True positive, true negative, false positive, and false negative data were extracted to calculate pooled sensitivity, specificity, positive and negative likelihood ratios, diagnostic odds ratio, and area under the receiver operating characteristics curve. The Deeks’ funnel plot was applied for publication bias assessment, Cochran’s Q test and I2 index were applied for inter-study heterogeneity assessment. Besides, a subgroup analysis was performed for determining the source of heterogeneity between studies. P value < 0.05 was considered significance. All analysis were done by STATA version 14.

**Results:**

CircRNA presented a sensitivity of 76% (95% confidence interval [95%CI]: 66-84%), specificity of 77% (95%CI: 58-89%), positive LR of 3.25 (95%CI: 1.69–6.23), negative LR of 0.31 (95%CI: 0.21–0.46), DOR of 10.41 (95%CI: 4.26–25.41), and AUC of 0.82 (95%CI: 0.79–0.85) for diabetes mellitus detection. More specifically, hsa_circ_0054633 showed a sensitivity of 67% (95%CI: 53-81%) and a specificity of 82% (95%CI: 63-100%).

**Conclusion:**

CircRNAs show highly accurate diagnostic capability for type 2 diabetes mellitus and gestational diabetes mellitus. High sensitivity of circRNAs introduces them as potential noninvasive biomarkers for early diagnosis of diabetes mellitus and their high specificity introduces them as potential therapeutic targets by regulation of their expression.

**Supplementary Information:**

The online version contains supplementary material available at 10.1186/s12920-023-01476-0.

## Introduction

Diabetes mellitus (DM) is a chronic and progressive condition defined by hyperglycemia caused by abnormalities in insulin production, insulin receptor sensitivity, or both [1]. Due to its long-term consequences, diabetes is now one of the leading causes of death in the world [2]. The International Expert Committee indicated that evaluating glycated hemoglobin (HbA1c) is a valid approach for diabetes diagnosis [3]. The World Health Organization (WHO) and the American Diabetes Federation stated that fasting blood glucose (FBG) and oral glucose tolerance test (OGTT) are both gold standards for diabetes diagnosis [4, 5]. Mechanistically, different molecular pathways and genetic factors are recognized to be related to some pathological conditions that cause hyperglycemia [6]. Several studies have documented that utilizing circRNAs can be effective in understanding the pathogenesis of various DM-related complications including diabetic retinopathy (DR), diabetic nephropathy (DN), and diabetic cardiomyopathy (DC) [7–9].Therefore, an accurate diagnosis of these associated molecules or genetic factors, along with the appropriate use of blood glucose tests and other potential factors influencing the disease progress, may lead to the development of comprehensive approaches for the early diagnosis of diabetes, and a guided treatment of DM and its complications [10, 11].

More recently, a great progress has been made towards the identification and employment of novel biomarkers to aid in the early detection of the disease, exploration of medical response, and the assessment of treatment benefits in a variety of diseases, including diabetes [12–14]. Various biomarkers including blood sugar, proteins, and particular nucleic acids can be employed as potential disease predictors [15]. Circular RNAs (circRNAs) are a new type of non-coding RNA molecules with a covalently closed loop that loses both polarity and a polyadenylated tail [16]. Circular RNAs are classified into three types: exonic circular RNA, intronic circular RNA, and intergenic circular RNA [17]. Exonic circular RNA is the most frequent type of circular RNA and usually is termed circRNA. The steadiness, abundancy, and evolutionary preservation of circRNAs through the species suggest that they could play a significant regulatory function [18]. Therefore, recent studies suggest that circRNAs may act as miRNA sponges because of the competitive endogenous RNA (ceRNA) system [19]. The remarkable biological stability of circRNAs makes it ideal for usage as a disease biomarker. The detection of circRNA in the blood stream has been associated with the control of insulin production as well as the development of diabetes [20]. The changing production of circRNA in the blood of type 2 diabetes patients has been confirmed, and some circRNAs such as hsa_circ_0054633 were recommended as possible diabetes diagnostic biomarkers [21]. Even though several studies have reported diagnostic value of circRNAs [22–26], the efficacy of circRNAs in the early detection of diabetes remains uncertain due to the inconsistency or heterogeneity in the reported performances of these studies. Thus, we conducted a comprehensive systematic review and meta-analysis of currently available data to investigate the accuracy of circRNAs in diabetes mellitus diagnosis.

## Methods

### Search strategy

We conducted a systematic review and meta-analysis in accordance with the Preferred Reporting Items for Systematic and Meta-analyses (PRISMA) statements, and registered its protocol in the International Prospective Register of Systematic Reviews (PROSPERO, CRD42022295996). We searched PubMed, Scopus, and Web of Science databases to retrieve all relevant studies investigating the diagnostic accuracy of circRNA for diabetes mellitus. All mentioned databases were searched without language restrictions to identify eligible studies based on the following main study keywords including “Diabetes”, “Circular RNA”, “Diagnostic Values”, and their synonyms. Gray Literature was then searched to access unpublished articles and dissertations or international reports. In addition, after the final selection of articles, a manual search was performed by reviewing the references of related articles. The search strategy in international databases was independently conducted by two researchers (HDB and PA) and the disputes were resolved by a third person (YM).

### Study selection

Eligible studies were independently selected by two reviewers (AH and PA) based on the following inclusion and exclusion criteria.

#### Inclusion criteria

All cohort studies, with no language restrictions, which have investigated the diagnostic accuracy of circRNA for diabetes mellitus were included. The population of interest was patients with diabetes mellitus including type 1 diabetes mellitus, type 2 diabetes mellitus, and gestational diabetes mellitus. The index test of interest was circRNAs. The reference test of interest was the gold standard laboratory tests for diabetes mellitus based on the American Diabetes Association (ADA) standards (FPG ≥ 126 mg/dL [7.0 mmol/L], 2-hour plasma glucose ≥ 200 mg/dL [11.1 mmol/L], HA1C ≥ 6.5%, and in a patient with classic symptoms of hyperglycemia or hyperglycemic crisis, a random plasma glucose ≥ 200 mg/dL [11.1 mmol/L]) (27). The target condition of interest was diagnostic performance of test including sensitivity and specificity.

#### Exclusion criteria

Case reports, case series, reviews, and meta-analyses were excluded. Studies in which false positive (FP), false negative (FN), true positive (TP), and true negative (TN) values of test were not reported or could not be calculated due to no data of sensitivity, specificity, the number of diabetic patients, and the number of healthy controls.

### Data extraction and quality assessment

Data extraction was independently performed by two reviewers (AH and PA). The following items were recorded: author, circRNA name, year, country, number of study center, study design, period of enrollment, reference standard for diabetes detection, number of patients, type of diabetes, sample source of circRNA, method of circRNA detection, reference gene, cutoff value regarding sensitivity and specificity Besides, two reviewers (AH and PA) independently evaluated the quality of studies according to the Quality Assessment of Diagnostic Accuracy Studies-2 (QUADAS-2) tool [28]. This tool investigated the risk of bias and concerns regarding the applicability of the included studies. Any discrepancies were resolved by a third investigator (HD).

### Statistical analysis

All statistical analyses were performed in STATA (version 14.1). First, TP, TN, FP, and FN values were extracted from the studies or calculated based on sensitivity, specificity, number of diabetic patients, and number of controls. For preservation of two-dimensional nature of original data, we took the advantage of bivariate random-effects model analysis (BRMA) following by hierarchical summary receiver operating characteristic (HSROC) model. Then, the MIDAS module was applied for determining the pooled diagnostic ability of circRNA including sensitivity, specificity, positive and negative likelihood ratios (LRs), diagnostic odds ratio (DOR), area under the receiver operating characteristics curve (AUC). The Deeks’ funnel plot was applied for publication bias assessment [29], and Cochran’s Q test and I^2^ index were applied for inter-study heterogeneity assessment. Besides, a subgroup analysis was performed for determining the source of heterogeneity between studies. Additionally, we sub-grouped the studies based on the circRNA (hsa_circ_0054633 vs. other circRNAs), study design (prospective vs. retrospective), number of participants (≥ 100 vs. < 100), publication year (≥ 2020 vs. < 2020), type of diabetes mellitus (Type 2 diabetes vs. gestational diabetes), the reference gene (beta actin vs. GAPDH). P value < 0.05 was considered significance.

## Results

### Literature search

**Fig. 1:**
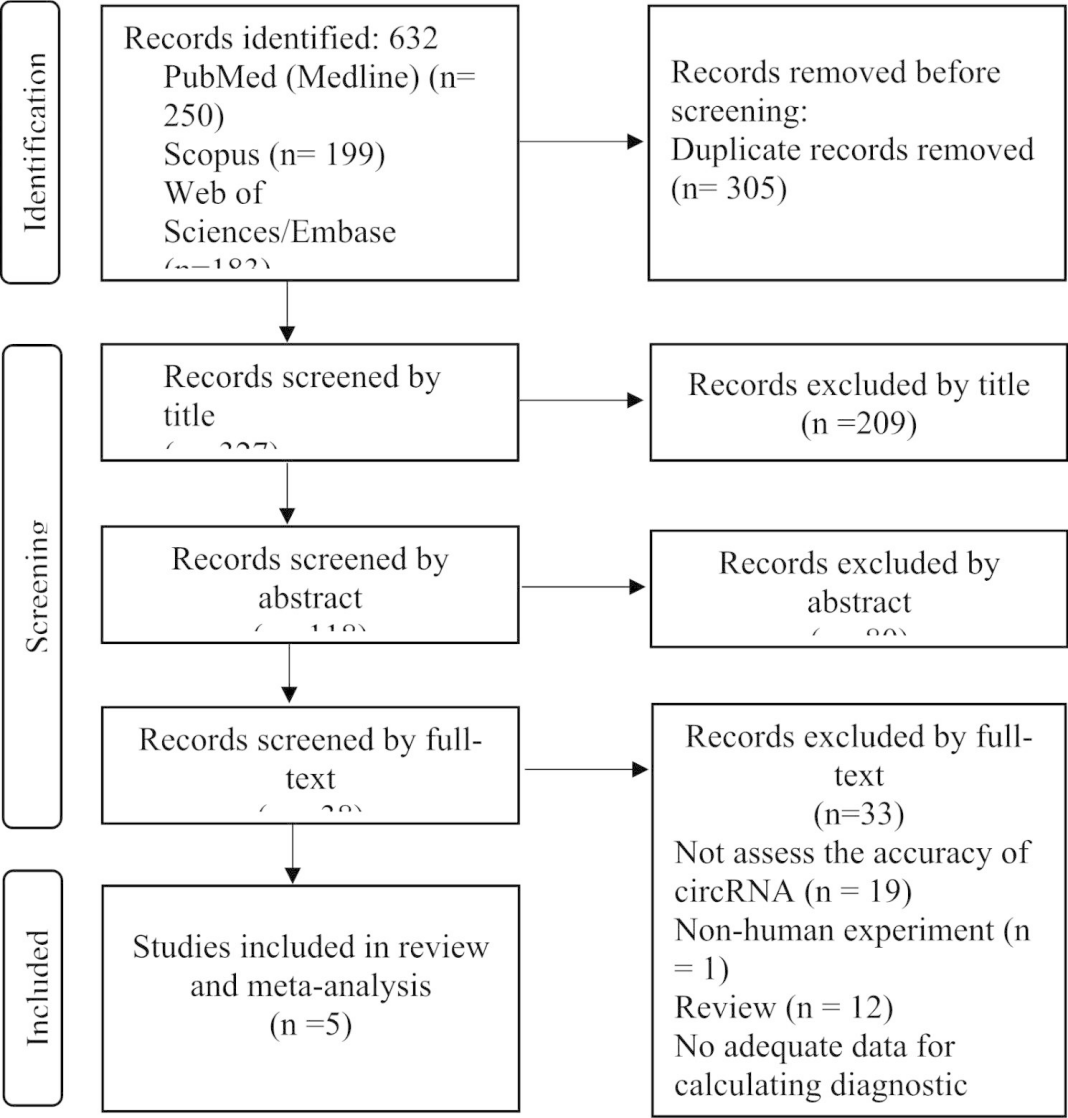
PRISMA chart of the included studies

The initial search yielded 632 results. After the removal of duplications, 327 articles remained and were screened based on their title and abstract. 289 articles were excluded because of irrelevancy, and 38 articles were screened with full-text. Finally, 5 studies [22–26] met all eligibility criteria and were considered for meta-analysis. Figure 1 shows the PRISMA flowchart of the literature search.

### General features of the included studies

Table 1 shows the baseline characteristics of the included studies. A total of 1284 participants (382 diabetic patients and 902 healthy individuals) were included in this meta-analysis. Study participants were enrolled from April 2015 to October 2019. Out of 5 studies, three [22, 24, 26] were prospective cohorts and two [23, 25] were retrospective cohorts. Type 2 diabetes patients were investigated in three of the studies [22, 25, 26], and gestational diabetes in the other two studies [23, 24]. Of note, based on our search strategy, we did not find any study on type 1 diabetes with use of circRNAs. Besides, hsa_circRNA_0054633 was investigated in three studies [22, 23, 25]. Additional file 1 shows the quality of included studies. All included studies had a low risk of bias regarding flow and timing, reference standard, and patient selection as well as low concerns of applicability regarding reference standard and patient selection. However, there were moderate concerns of applicability regarding the index test.

**Table 1:** Baseline characteristics of included studies

Author, year country	Study design No. of centers	Period of enrollment	Sample size (DM / non-DM)	Type of diabetes	Sample source	Detection method	Reference Gene	circRNA Name
Zhao Z. et al. 2016 (22), China	Prospective cohort, Single-center	July 2015 - June 2016	64/60	T2DM	whole blood	qRT PCR (SYBR)	NR	hsa_circ_0054633 hsa_circ_0068087
Wu H. et al. 2019 (23), China	Retrospective Cohort, Single-center	July 2017 - February 2018	65/65	GDM	Maternal serum in the second and third trimester, umbilical cord blood	qRT PCR (SYBR)	Beta actin	hsa_circ_0054633 hsa_circ_063981 hsa_circ_102682 hsa_circ_103410
Yang H et al. 2020 (24), China	Prospective cohort, Single-center	April 2015 - July 2016	106/ 630	GDM	Plasma	GoTaq qPCR Master Mix (Promega) on a ViiA 7 Real-time PCR System (Applied Biosystems)	NR	hsa_circ_102893
Liang H. et al. 2021 (25), China	Retrospective Cohort, Single-center	January 2018 - March 2019	44/44	T2DM	Serum	qRT PCR (SYBR)	NR	hsa_circ_0054633
Yingying Z. et al. 2021 (26), China	Prospective cohort, Single-center	January 2019 - October 2019	103/103	T2DM	whole blood	Both the Prime Script™ RT reagent Kit with gDNA Eraser and Q-PCR kits were equipped with Takara Bio Inc, Japan	GAPDH	hsa_circ_0071106

GDM: gestational diabetes mellitus, T2DM: type 2 diabetes mellitus, DM: diabetes mellitus, NR: Not Reported

### Diagnostic accuracy of circRNA for diabetes mellitus

**Fig. 2:**
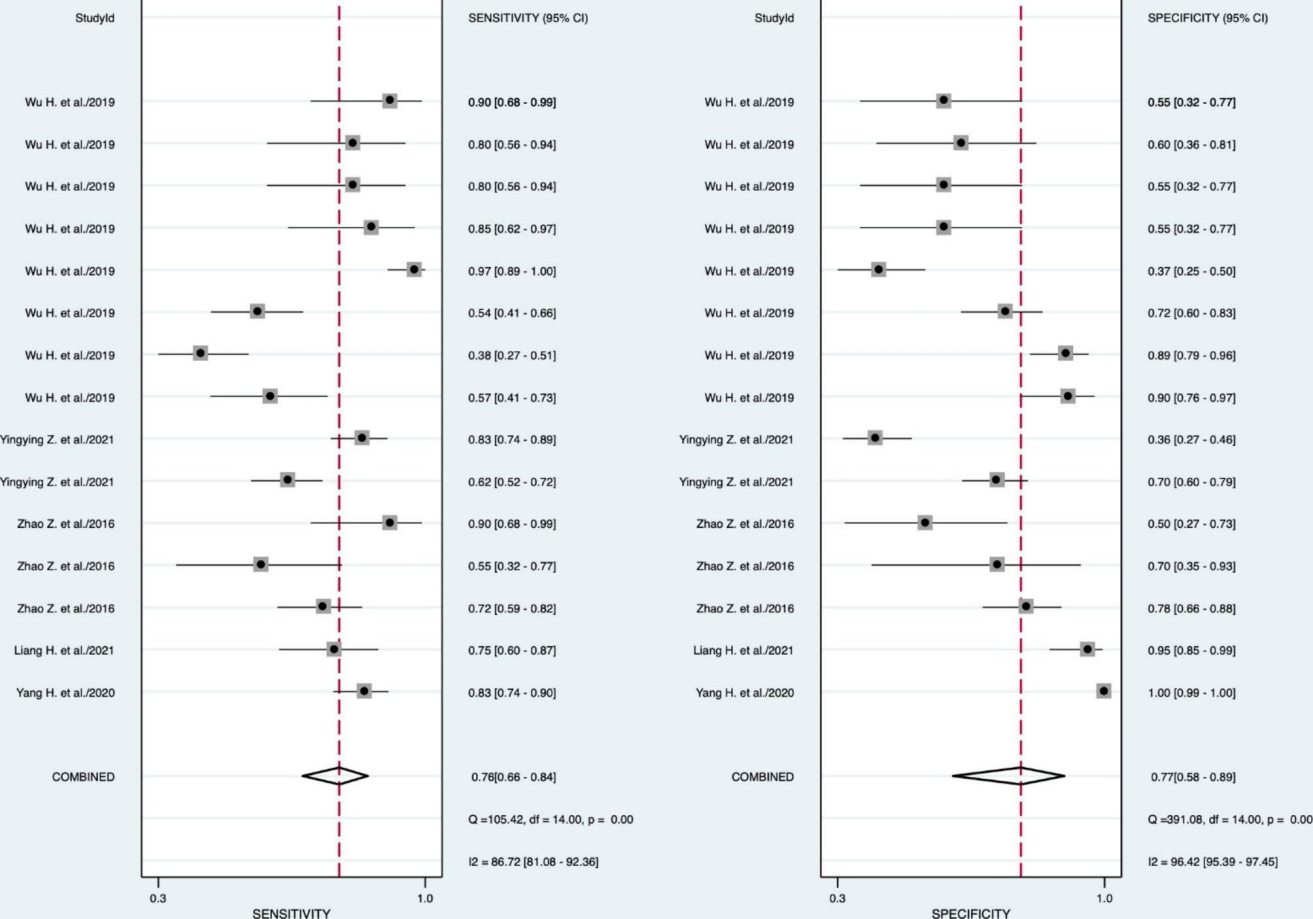
The pooled sensitivity (**a**) and pooled specificity (**b**) of circRNA for diabetes mellitus detection

**Fig. 3:**
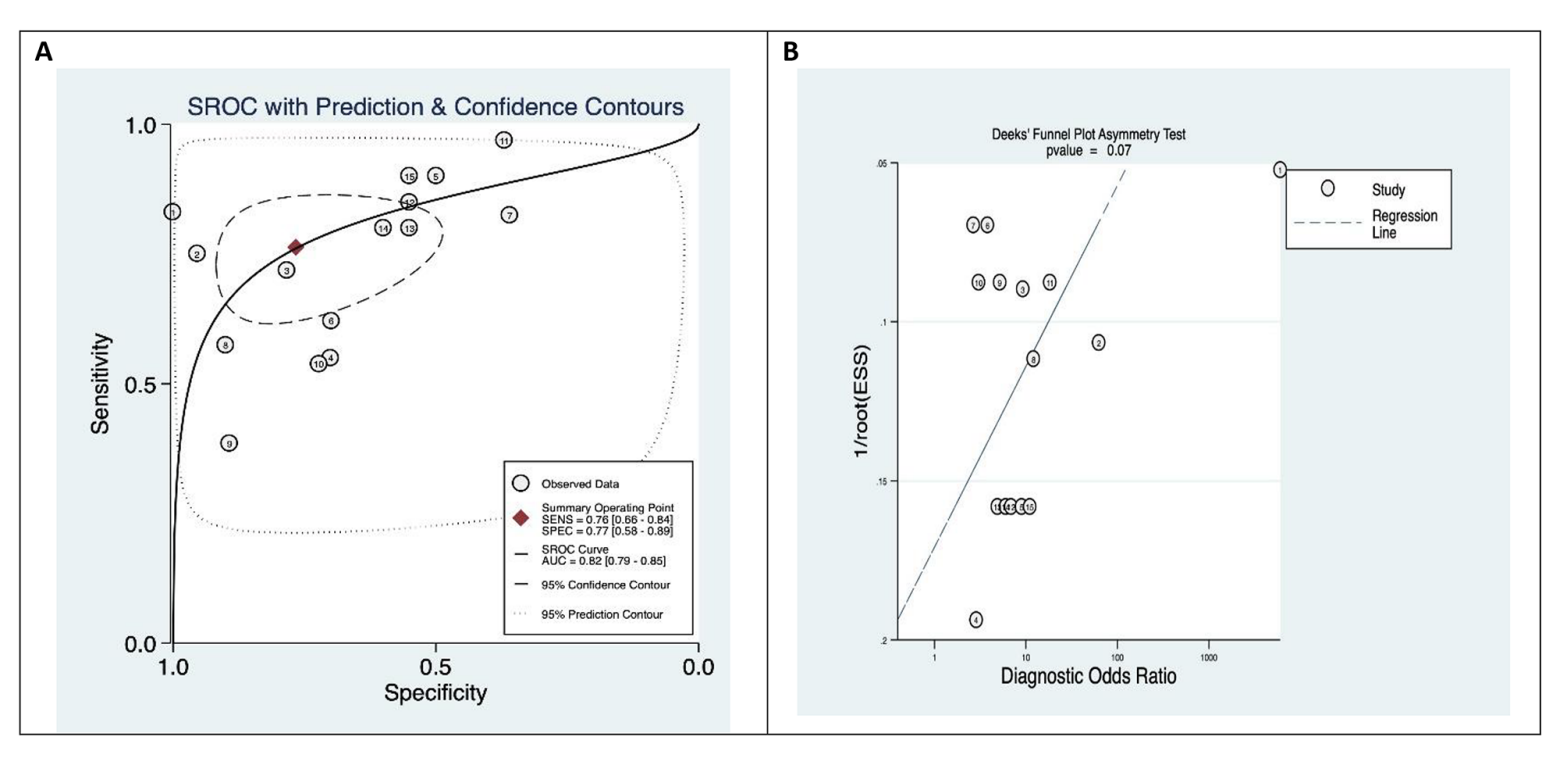
**(a)** The receiver operating characteristic (ROC) curve of circRNA for diabetes mellitus detection, **(b)** The Deek’s funnel-plot asymmetry test of circRNA for diabetes mellitus detection. The horizontal axis represents the diagnostic odds ratio (DOR) as an indicator of the diagnostic accuracy, and the vertical axis represents the inverse of the square root of the effective sample size (1/root (ESS)). This figure shows symmetrical effect size measures (DOR) over different sample sizes, indicating no presence of publication bias

The pooled analyses on sensitivity and specificity of circRNA are depicted in Fig. 2a and b, respectively. CircRNAs had pooled sensitivity of 76% (95% CI: 66–84%; Cochran’s Q: 105.42; I2 index: 86.72%) and pooled specificity of 77% (95% CI: 58–89%; Cochran’s Q: 391.08; I2 index: 96.42%) for diagnosis of diabetes mellitus. The ROC analysis (Fig. 3a) shows high accuracy (AUC = 0.82; 95% CI: 0.79–0.85) for circRNA as a factor for diagnosis of diabetes mellitus. The pooled positive LR was estimated as 3.25 (95% CI: 1.69–6.23; Cochran’s Q: 158.06; I^2^ index = 87.80%), the pooled negative LR was estimated as 0.31 (95% CI: 0.21–0.46; Cochran’s Q: 85.34; I^2^ index = 83.60%; Additional file 2a and 2b, respectively), the pooled diagnostic score was estimated as 2.34 (95% CI: 1.45–3.24; Cochran’s Q: 55.47; I^2^ index = 74.76%), and the pooled DOR was estimated as 10.41 (95% CI: 4.26–25.41; Cochran’s Q: 2.2e + 9; I^2^ index = 100%; Additional file 3a and 3b, respectively).

### Publication bias assessment

Figure 3b shows the Deeks’ funnel plot asymmetry test, assessing the presence of publication bias in this meta-analysis. The X-axis represents DOR as an indicator of the diagnostic accuracy, and the Y-axis represents the inverse of the square root of the effective sample size (1/root (ESS)). Through a qualitative assessment of publication bias, we found a symmetrical effect size measures (DOR) over different sample sizes (1/root (ESS)), indicating publication bias is unlikely. Besides, the quantitative assessment of publication bias through Egger’s regression line displays a p-value of 0.07, confirming the absence of publication bias.

### Subgroup analysis

The subgroup analysis showed that type of circRNA was the source of heterogeneity for sensitivity between studies. For instance, hsa_circRNA_0054633 had a sensitivity of 67% (95% CI: 53–81%) while other types of circRNA had a sensitivity of 82% (95% CI: 73–91%; p value = 0.01). Besides, we found that study design, sample size of studies, publication year, type of diabetes mellitus, reference gene did not influence the sensitivity and specificity of circRNA for diabetes mellitus detection. The meta-regression and subgroup results are shown in Table 2.

**Table 2:** Subgroup analysis of the diagnostic performance of circRNA for diabetes diagnosis

Subgroups	Covariates	No. of studies	No. of reports on circRNA accuracy	Pooled sensitivity [95% CI]	P value	Pooled specificity [95% CI]	P value
**Type of circRNA**	hsa_circ_0054633	3	7	0.67 [0.53–0.81]	0.01	0.82 [0.63–1.00]	0.65
	Other types	4	8	0.82 [0.73–0.91]		0.71 [0.47–0.95]	
**Study design**	Retrospective	2	9	0.76 [0.64–0.88]	0.25	0.73 [0.51–0.95]	0.70
	Prospective	3	6	0.76 [0.63–0.90]		0.81 [0.60–1.00]	
**Sample size**	≥ 100	4	7	0.74 [0.61–0.87]	0.11	0.81 [0.62–1.00]	0.66
	< 100	3	8	0.78 [0.67–0.90]		0.71 [0.47–0.96]	
**Year of publication**	≥ 2020	3	4	0.77 [0.61–0.92]	0.35	0.91 [0.79–1.00]	0.11
	< 2020	2	11	0.76 [0.65–0.86]		0.68 [0.47–0.88]	
**Type of diabetes**	GDM	2	9	0.77 [0.66–0.89]	0.39	0.80 [0.61–0.98]	0.73
	T2DM	3	6	0.74 [0.60–0.89]		0.71 [0.43–0.99]	
**Reference Gene**	beta Actin	1	8	0.76 [0.62–0.89]	0.81	0.66 [0.53–0.80]	0.62
	GAPDH	1	2	0.74 [0.49–1.00]		0.53 [0.25–0.82]	

GDM: gestational diabetes mellitus, T2DM: type 2 diabetes mellitus

## Discussion

To the best of our knowledge, this is the first meta-analysis that is particularly directed to evaluate the diagnostic value of circRNAs for diabetes mellitus. We have screened a total of five cohort studies and included 1284 participants including 382 diabetic patients and 902 healthy individuals in this systematic review and meta-analysis. The quality of the five included studies was relatively high with no significant publication bias. The overall performance based on the pooled results demonstrated pooled sensitivity of 76%, pooled specificity of 77%, and AUC of 0.82. Based on subgroup analyses, the circRNA54633 was less sensitive than other types of circRNAs (has_circ_0068087, 0054633, 063981, 102,682, 103,410, 102,893, 0054633, 0071106). The pooled positive LR of 3 indicates that diabetic individuals had a 3-fold greater possibility of dysregulated expression of circRNAs than healthy controls. Similarly, a pooled negative LR of 0.31 suggests that individuals with normal expression of circRNAs have a 31% chance of having diabetes. The pooled diagnostic statistics showed moderate diagnostic tool accuracy suggesting that circRNAs have enough statistical power to distinguish diabetic patients from non-diabetic individuals regardless of the type of diabetes and the target gene.

Prior studies suggested the role of circRNAs in the pathogenesis of diabetes mellitus [30]. Thousands of circRNAs are expressed in human pancreatic islets [31]. CircRNAs (CiRS-7 and circHIPK3) were shown to enhance β-cell function and improve insulin granule secretion by microRNA sponging activities [20, 31]. Evidence from included studies revealed different expression levels of distinct circRNAs were detected in serum, plasma, or umbilical cord blood of patients with type 2 diabetes mellitus and pregnant women with gestational diabetes compared to healthy controls [22–26]. For instance, the expression levels of hsa_circRNA_0054633 [23], hsa_circ_0054633 [25], hsa_circ_0071106, hsa_circ_0071271, and hsa_circ_0000284 [26] were found increased in the circulation in diabetic patients. While the hsa_circRNA_102893 [24] was downregulated in women with gestational diabetes. Bioinformatics analysis showed that downregulation of hsa_circRNA_102893 induces upregulation of hsa- miR-197-3p [32], and the level of hsa-miR-197-3p was associated with impaired β-cell function and insulin resistance in diabetes mellitus [33].

Nevertheless, these findings should be interpreted with caution since all the included studies have been exclusively performed in China and this may result in population selection bias. More investigation on greater geographical and ethnic diversity among participants is highly recommended. Additionally, there were some restrictions in the “index test” due to not specified cut-off points. More precisely, the study by Wu H et al. [23] did not report the cut-off points at which sensitivity and specificity were calculated for hsa_circ_0054633, hsa_circ_063981, hsa_circ_102682, and hsa_circ_103410 in detecting diabetes mellitus. Other limitations include the limited number of enrolled studies, the small sample size of some of the studies, and considerable heterogeneity between studies. Likewise, some confounding information including age, gender proportion, and physical health of participants was present. Owing to the restrictions of this meta-analysis, high-quality multi-center prospective studies with larger sample sizes are warranted to further validate the clinical application of circRNAs as a diagnostic biomarker of diabetes mellitus.

Despite its limitations, this work offers a comprehensive analysis of a panel of circRNAs as a diagnostic tool for the early detection of diabetes mellitus. Future well-designed research using a combination of circRNAs, or in combination with microRNAs and other clinical diagnostic biomarkers would improve the accuracy of the test as a biomarker of prognosis, diagnosis, or treatment of diabetes mellitus.

## Conclusion

CircRNAs are identified as highly accurate diagnostic biomarkers for type 2 diabetic mellitus and gestational diabetes mellitus. The high sensitivity of these circRNAs introduces them as good biomarkers for early detection of diabetes mellitus and high specificity of these circRNAs introduces them as good therapeutic targets for the management of diabetes mellitus through the regulation of these circRNAs expression. This study suggests the possibility of the utility of circRNAs as potential noninvasive biomarkers for better understanding of the diabetes mellitus pathogenesis.

## Electronic supplementary material

Below is the link to the electronic supplementary material.

Supplementary Material 1

Supplementary Material 2

## Data Availability

All data generated or analyzed during this study are included in this published article.

## References

[1] Banday MZ, Sameer AS, Nissar S (2020). Pathophysiology of diabetes: an overview. Avicenna J Med.

[2] Dehghanbanadaki H, Aazami H, Ejtahed HS, Sohrabi A, Raftar SKA, Tarashi S (2022). The global scientific publications on gut microbiota in type 2 diabetes; a bibliometric, scientometric, and descriptive analysis. J Diabetes Metabolic Disorders.

[3] International Expert Committee report on the (2009). Role of the A1C assay in the diagnosis of diabetes. Diabetes Care.

[4] World Health Organization. Use of glycated haemoglobin (HbA1c) in diagnosis of diabetes mellitus: abbreviated report of a WHO consultation. World Health Organization; 2011. 26158184

[5] American Diabetes Association (2017). 2. Classification and diagnosis of diabetes. Diabetes Care.

[6] American Diabetes Association (2014). Diagnosis and classification of diabetes mellitus. Diabetes Care.

[7] Fan W, Pang H, Xie Z, Huang G, Zhou ZJFiE.Circular RNAs in diabetes mellitus and its complications. 2022;13. 10.3389/fendo.2022.885650 PMC9376240 35979435

[8] Crawford TN, Alfaro DV, Kerrison JB (2009). Jablon EPJCdr. Diabet retinopathy angiogenesis.

[9] Xu B, Wang Q, Li W, Xia L, Ge X, Shen L et al. Circular RNA circEIF4G2 aggravates renal fibrosis in diabetic nephropathy by sponging miR-218. 2022;26(6):1799–805. 10.1111/jcmm.16129 PMC8918410 33615661

[10] Will new diagnostic criteria for diabetes mellitus change phenotype of patients with diabetes? Reanalysis of European epidemiological data. DECODE Study Group on behalf of the European Diabetes Epidemiology Study Group (1998). BMJ (Clinical research ed).

[11] Wang J (2006). Electrochemical biosensors: towards point-of-care cancer diagnostics. Biosens Bioelectron.

[12] Dehghanbanadaki H, Aazami H, Razi F, Nasli-Esfahani E, Norouzi P, Hashemi E (2022). The global trend of exosome in diabetes research: a bibliometric approach. Diabetes & Metabolic Syndrome: Clinical Research & Reviews.

[13] Hosseinkhani S, Aazami H, Hashemi E, Dehghanbanadaki H, Adibi-Motlagh B, Razi F (2021). The trend in application of omics in type 2 diabetes researches; a bibliometric study. Diabetes & Metabolic Syndrome: Clinical Research & Reviews.

[14] Hosseinkhani S, Dehghanbanadaki H, Aazami H, Pasalar P, Asadi M, Razi F (2021). Association of circulating omega 3, 6 and 9 fatty acids with gestational diabetes mellitus: a systematic review. BMC Endocr Disorders.

[15] Marrugo-Ramírez J, Mir M, Samitier J. Blood-Based Cancer Biomarkers in Liquid Biopsy: A Promising Non-Invasive Alternative to Tissue Biopsy. International journal of molecular sciences. 2018;19(10). 10.3390/ijms19102877 PMC6213360 30248975

[16] Chen Z, Zhang L, Han G, Zuo X, Zhang Y, Zhu Q (2018). A Meta-analysis of the diagnostic accuracy of circular RNAs in Digestive System Malignancy. Cell Physiol Biochem.

[17] Lasda E, Parker R. Circular RNAs: diversity of form and function. RNA (New York, NY). 2014;20(12):1829-42. 10.1261/rna.047126.114 PMC4238349 25404635

[18] Shao T, Pan Y-h (2021). Xiong X-d. Circular RNA: an important player with multiple facets to regulate its parental gene expression. Mol Therapy-Nucleic Acids.

[19] Barrett SP, Salzman J (2016). Circular RNAs: analysis, expression and potential functions. Development.

[20] Xu H, Guo S, Li W, Yu P (2015). The circular RNA Cdr1as, via miR-7 and its targets, regulates insulin transcription and secretion in islet cells. Sci Rep.

[21] Zhang Z, Yang T, Xiao J (2018). Circular RNAs: promising biomarkers for Human Diseases. EBioMedicine.

[22] Zhao Z, Li X, Jian D, Hao P, Rao L, Li M (2017). Hsa_circ_0054633 in peripheral blood can be used as a diagnostic biomarker of pre-diabetes and type 2 diabetes mellitus. Acta Diabetol.

[23] Wu H, Wu S, Zhu Y, Ye M, Shen J, Liu Y (2019). Hsa_circRNA_0054633 is highly expressed in gestational diabetes mellitus and closely related to glycosylation index. Clin epigenetics.

[24] Yang H, Ye W, Chen R, Zeng F, Long Y, Zhang X (2020). Circulating expression of Hsa_circRNA_102893 contributes to early gestational diabetes mellitus detection. Sci Rep.

[25] Liang H, Hou L, Wang Q, Zhou X, Sha L, Xu L (2021). Serum hsa_circ_0054633 is elevated and correlated with clinical features in type 2 diabetes Mellitus. Annals of Clinical & Laboratory Science.

[26] Yingying Z, Yongji Y, Qiuting C, Rifang L, Zhuanping Z (2021). has_circ_0071106 can be used as a diagnostic marker for type 2 diabetes. Int J Med Sci.

[27] American Diabetes Association. 2. Classification and diagnosis of diabetes: standards of medical care in diabetes—2020. Diabetes care. 2020;43(Supplement 1):S14-S31. 10.2337/dc20-S002 31862745

[28] Whiting PF, Rutjes AW, Westwood ME, Mallett S, Deeks JJ, Reitsma JB (2011). QUADAS-2: a revised tool for the quality assessment of diagnostic accuracy studies. Ann Intern Med.

[29] Deeks JJ, Macaskill P, Irwig L (2005). The performance of tests of publication bias and other sample size effects in systematic reviews of diagnostic test accuracy was assessed. J Clin Epidemiol.

[30] Zaiou M (2020). circRNAs signature as potential diagnostic and prognostic biomarker for diabetes mellitus and related cardiovascular complications. Cells.

[31] Stoll L, Sobel J, Rodriguez-Trejo A, Guay C, Lee K, Venø MT (2018). Circular RNAs as novel regulators of β-cell functions in normal and disease conditions. Mol metabolism.

[32] Horton JD, Goldstein JL, Brown MS (2002). SREBPs: activators of the complete program of cholesterol and fatty acid synthesis in the liver. J Clin Investig.

[33] Zampetaki A, Kiechl S, Drozdov I, Willeit P, Mayr U, Prokopi M (2010). Plasma microRNA profiling reveals loss of endothelial miR-126 and other microRNAs in type 2 diabetes. Circul Res.

