# Supplementary material for: Diagnostic accuracy of circular RNA for diabetes Mellitus: a systematic review and diagnostic Meta-analysis
Source: BMC Med Genomics. 2023 Mar 8;16:48. doi: 10.1186/s12920-023-01476-0 (PMC9993609; doi:10.1186/s12920-023-01476-0)
Supplement: Supplementary file 2 — Supplementary Material 2 [file 12920_2023_1476_MOESM2_ESM.docx]

Additional file 1. Quality assessment including risk of bias and concerns regarding applicability of included studies.


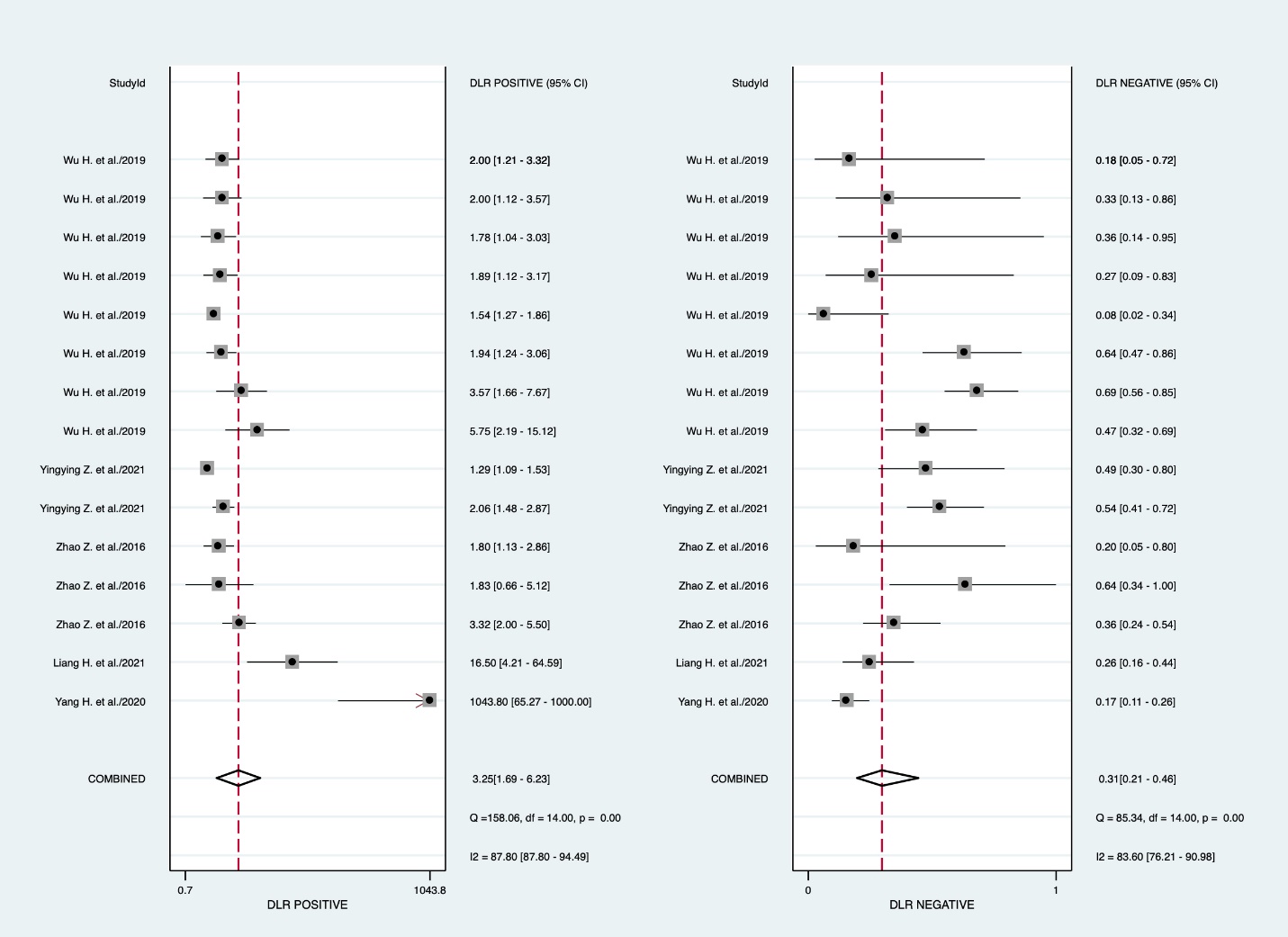


Additional file 2. The pooled positive likelihood ratio (a) and the pooled negative likelihood ratio (b) of circRNA for diabetes mellitus detection.


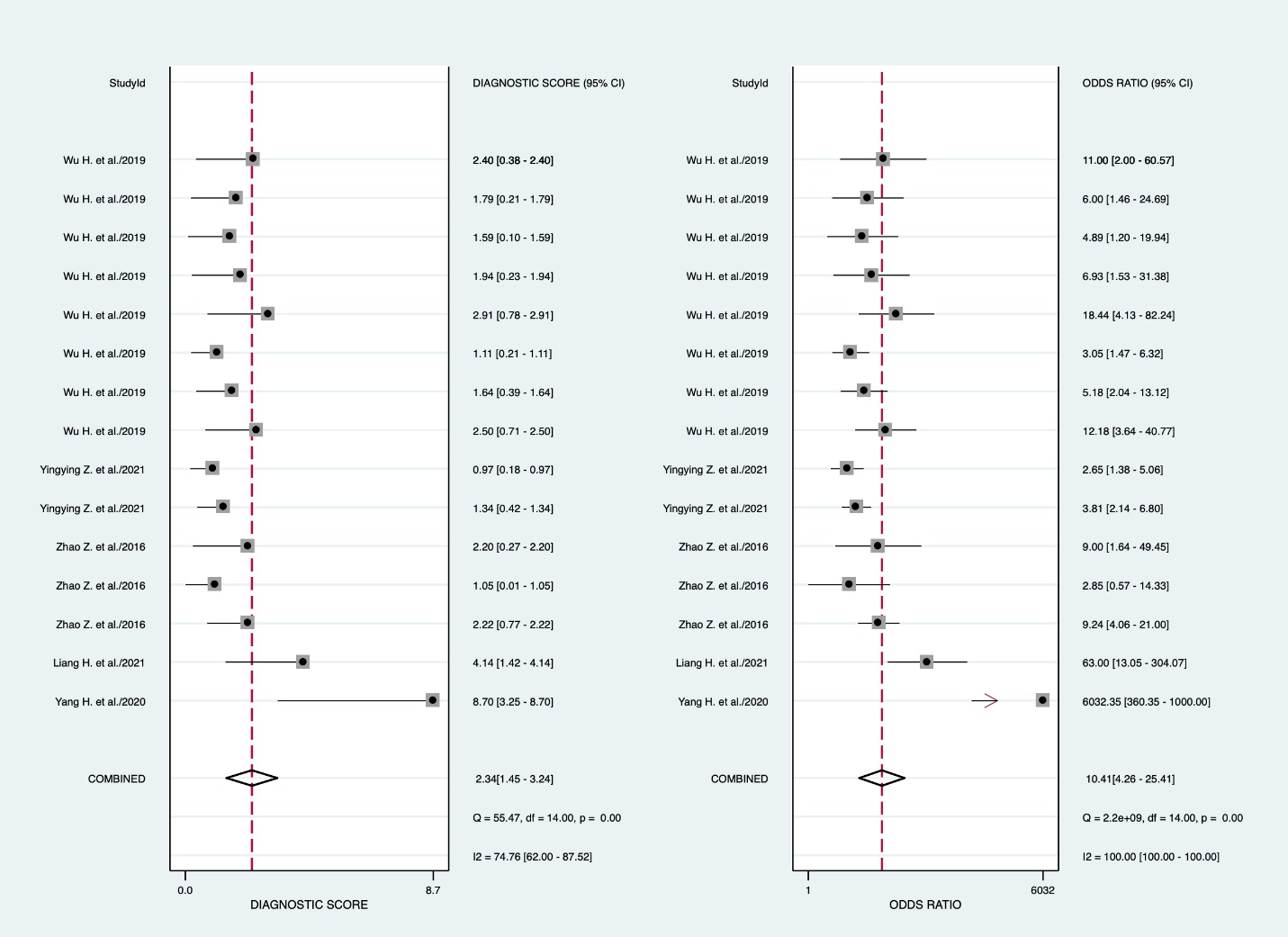


Additional file 3. The pooled diagnostic score (a) and the pooled diagnostic odds ratio (b) of circRNA for diabetes mellitus detection.
